# Therapeutic Notes

**Published:** 1896-08-15

**Authors:** 


### THERAPEUTIC NOTES.

### SO-CALLED CHOLAGOGUES.

If the views lately expressed by E. Stadelmann are correct, it is quite certain that we must modify our views on the subject of cholagogues. The definition of a cholagogue is that which causes an increased flow of bile. Cholagogues may, secondarily, be divided into direct and indirect. The direct are supposed to act directly on the liver cells and gall bladder, and include such drugs as podophyllin, rhubarb, sulphate of soda. The indirect sweep out of the intestine such bile as is therein contained, and thus indirectly stimulate a fresh secretion. In a most elaborate paper Stadelmann[1] reviews all the evidence that has accumulated on the subject, and not only criticises the methods which have been used to obtain it, but further rejects it as almost worthless. All experiments on the subject have been carried out on animals with biliary fistulæ, and the usual method is to give an animal a dose of some drug, and watch the resultant outflow of bile. If the drug causes an increased flow, it is usually called a cholagogue. Stadelmann's experiments have extended over years, and numbers of his pupils have assisted in the research. Dogs have always been used; their diet has been constant, and their body weight maintained at the same point, and the observation with the different drugs made over and over again under constant conditions. The result has been to show that water, alkaline salts, the usual drastic cholagogues, calomel, alcohol, and olive oil had no effect on the biliary secretion; while the effect of antipyrine, antifebrin, caffein, diuretics, santonin was doubtful. And of all the substances tried there were only two with any effect whatsoever, namely, salicylate of soda, and the bile salts. In the former case the fluid amount of bile only was increased, the solids remaining constant; in the case of the bile salts the solids, as well as the fluids, were increased. From a practical point of view Stadelmann denies the use of reputed cholagogues, or even true cholagogues, such as above described, in cases of biliary calculus or catarrhal jaundice; and if alkalies, water, and olive oil are of value in such cases it is not because of their cholagogue action, but from some other and not yet obvious cause.

[1] Berliner Klin. Woch., Nos. 9 and 10, 1896.

### EUCAÏNE, A NEW SUBSTITUTE FOR COCAÏNE.

In a paper read before the Hufeland Society (Hufeland'sch Gesellshaft), in Berlin, Vinci[1] gave a full account of the experiments made with this new drug in Professor Liebriech's laboratory. Eucaïne is itself a base of a somewhat insoluble nature; the hydrochlorate which, for many reasons, seems a more suitable salt for general purposes, is much more soluble in aqueous media, and may be used for producing local anæsthesia in solutions of 2 per cent. strength. It may, however, be employed in 5 per cent. strength. The 2 per cent. solution will produce complete local anæsthesia in three minutes, and the effects last for nearly half an hour. The general effect on the cardio-vascular system appears to be less noxious than that of cocaïne, inasmuch as, although the frequency of pulse is decreased, the blood pressure is not increased, and for this reason is less likely to produce syncopal effects. In its local effects it differs from cocaïne in that it produces anæsthesia with hyperæmia, instead of producing a condition of ischœmia, as is the case with the latter body. When used for ophthalmic purposes it has the further advantage of not interfering with the action of the muscles of accommodation. In a recent paper by Berger[2] several further points are noticed. He mentions that it may either be obtained in a crystalline form, methyl alcoholic solution, or in the form of scales from aqueous solution. That latter is devoid of certain irritating properties which attach to the crystalline variety. With regard to ophthalmic use, Berger points out that it has no effect on the size of the pupil; but he asserts that when dropped into the eye in full strength (3 per cent.) it at first causes a stinging sensation. To avoid this he recommends a 1 per cent. solution to start with, which is to be increased after a few minutes to the full strength. With ordinary cocaïne, owing to the constriction of the vessels which it causes, and the consequent ischæma, Berger has noticed a tendency to corneal ulceration or sloughing. With eucaïne, on the other hand, there is a tendency to capillary dilation, which, however, he asserts, never interferes with surgical operations in the eye. But by mixing eucaïne and cocaïne in equal parts the two effects are neutralised, and for certain purposes such a mixture may prove of use. Eucaïne can be sterilised before use by boiling without undergoing decomposition, and this Berger considers to be one of the most valuable properties of the new body. Brudenell Carter[3] also speaks highly of this new drug.

[1] Revue de Therapeutic, 1896, p 317. [2] Revue de Therapeutic, June 1, 1896, p. 355. [3] Lancet, July 11th, 1896.

